# Evaluation of voice commands for mode change in virtual reality implant planning procedure

**DOI:** 10.1007/s11548-022-02685-1

**Published:** 2022-06-15

**Authors:** Hanna-Riikka Rantamaa, Jari Kangas, Maarit Jordan, Helena Mehtonen, John Mäkelä, Kimmo Ronkainen, Markku Turunen, Osku Sundqvist, Ismo Syrjä, Jorma Järnstedt, Roope Raisamo

**Affiliations:** 1 Faculty of Information Technology and Communication Sciences, Tampere University, Kalevantie 4, 33014 Tampere, Finland; 2 Medical Imaging Centre, Department of Radiology, Tampere University Hospital, Teiskontie 35, 33520 Tampere, Finland; 3 Planmeca, Asentajankatu 6, 00880 Helsinki, Finland

**Keywords:** Virtual dental implant planning, Virtual reality, Dental implant, 3D imaging

## Abstract

**Purpose:**

In dental implantology, the optimal placement of dental implants is important to meet functional and aesthetic requirements. Planning dental implants in virtual three-dimensional (3D) environment is possible using virtual reality (VR) technologies. The three-dimensional stereoscopic virtual reality environment offers advantages over three-dimensional projection on a two-dimensional display. The use of voice commands in virtual reality environment to replace button presses and other simple actions frees the user’s hands and eyes for other tasks.

**Methods:**

Six dentomaxillofacial radiologists experimented using a prototype version of a three-dimensional virtual reality implant planning tool, and used two different tool selection methods, using either only button presses or also voice commands. We collected objective measurements of the results and subjective data of the participant experience to compare the two conditions.

**Results:**

The tool was approved by the experts and they were able to do the multiple-implant planning satisfactorily. The radiologists liked the possibility to use the voice commands. Most of the radiologists were willing to use the tool as part of their daily work routines.

**Conclusion:**

The voice commands were useful, natural, and accurate for mode change, and they could be expanded to other tasks. Button presses and the voice commands should be both available and used in parallel. The input methods can be further improved based on the expert comments.

## Introduction

The dental implant placement is important to meet functional and aesthetic requirements. Using virtual reality (VR) technologies the dental implant planning can be done in virtual three-dimensional (3D) environment. Cone-beam computed tomography (CBCT) images are taken from the patient’s operative area and used for virtual implant planning. The planning can also be done using more traditional way, viewing and processing the models on 2D displays. In clinical practice for diagnosis, radiologists and surgeons typically evaluate the 3D data on 2D displays in three different directions using layers (axial, coronal, and sagittal) [1], and in implant planning utilizing special implant planning software and cross-sectional slices.

In VR, the 3D models can be manipulated and translated freely. Seeing the 3D object from three angles at a time allows for precise manipulation but requires great spatial understanding of the structure [2]. Viewing 3D data in a 3D environment would provide an advantage over 2D displays for perception and understanding (Sutherland [3]). VR has been used in medicine and dentistry [4–7]. There are a great number of ways to interact in VR and it is crucial to develop the most suitable ones.

The user interface components are not used similarly in VR and in 2D graphical user interface. Familiar 2D interface elements, like buttons and menus, must be re-designed. The use of voice commands in VR has been studied with and without other interaction methods [8]. Voice is an interesting modality because it frees the user’s hand to other, more important tasks [8, 9].

**Fig. 1:**
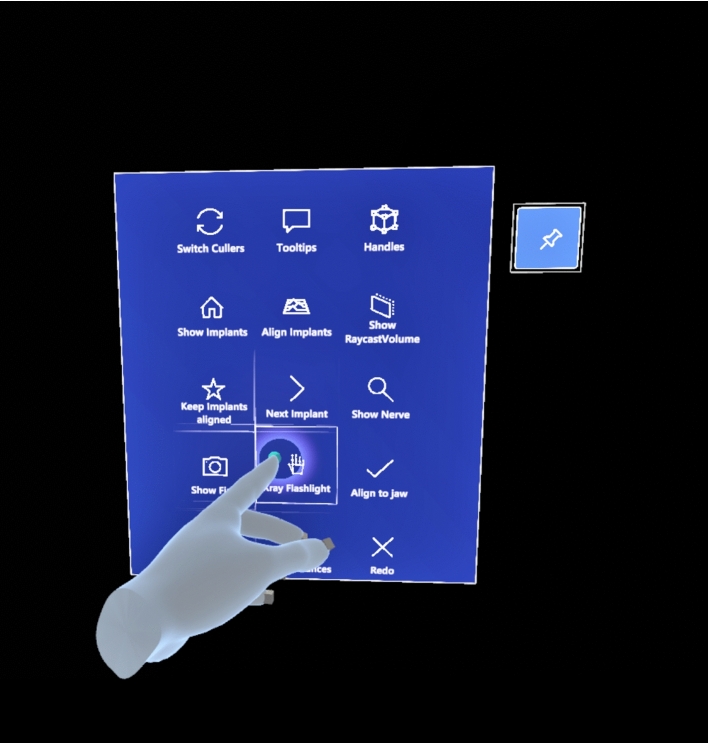
A tool is selected from a menu. The selection can be made by pressing the button by a (virtual) finger, by pointing a ray and pressing a trigger, or by a voice command, talking aloud the name of the selection visible on the menu

We compared two techniques to execute mode or tool changes. The options were either to make the change by tapping separate buttons on a virtual mid-air menu by the controller, see Fig. 1, or by using short voice commands (having the button presses as a backup). The aim was to test the voice commands by radiologists while having hand pointing as a comparison. While we collected objective measures of the implant planning, the main measurements were the subjective evaluations. Four out of the six participants in our study were using the VR based implant planning system for the second time, while two participants were first time users. In the experiment, the participants were asked to make three implant plans.

The participants liked the system and most were willing to use it daily as a part of their work routines. Both conditions had advantages and disadvantages, hand pointing and the voice commands should be both available and used in parallel. The voice commands can make the use of the system efficient, decreasing the need for turning around in the VR environment, and they were natural to use.

## Background

In dental implantology, one or multiple teeth are replaced with the dental implants and prosthesis, an implant supported denture or various fixed supra-structures such as crowns or dental bridges. When placing the implants, it is essential to pay attention to the surrounding anatomical structures. Complications of implant surgery can be temporary or permanent nerve injury and chronic pain, bleeding—sometimes even life-threatening hemorrhages, malposition or dislocation of implants, injury to adjacent teeth and fracture of the mandible [10–14].

The 3D cone-beam computed tomography (CBCT) data and 2D X-ray data are both used in implant planning process. When a challenging anatomy is in question, 3D imaging and preoperative planning of implantation with CBCT-scan is helpful. Many of the complications can be avoided by preoperative planning [10]. The dental professional sees operative area like the jaw with teeth, roots, and mandibular canals from the data and needs to define the correct size and location of the implant based on the data. A safety margin must be left between the implant and the nearest teeth as well as between the implant and various anatomical structures.

### Mode changing using a mid-air menu in VR

In most cases, some explicit act is used for mode changing [15], like a voice command or a press of a menu button. Menus are used to issue commands, begin dialog sequences, change the mode of interaction etc. [16]. Wang et al. [17] stated that the performance of space fixed menus (in VR) was better than handheld menus. Hand pointing with button press would be the most suitable interaction technique for space fixed menu [17]. Ray-casting allows users to select objects beyond their area of reach and require less physical movement even in their area of reach. The user points at the objects with a virtual ray which is usually attached to a tracked controller [17].

### Voice commands

The use of speech interfaces is growing in popularity [18]. Voice as an interaction method frees the user’s hands and eyes for other tasks [8, 9, 19, 20].

Using voice is problematic when defining directions, distances, and spatial relations [21], while voice is highly efficient when activating uniquely defined, simple commands. Known limitations for the voice use would be, for example, environmental disturbances, like noise that would affect the recognition results. Voice input is convenient and efficient in that it enables selecting a specific command from a large set with a single act (vs. a menu system often required when using pointing devices). Using voice for selection also allows the users to keep their attention on the task without looking the menu. Voice can also be used in multimodal systems. The combination of voice and pointing gestures is a natural match (e.g., “Put That There” system (Bolt et al. [22])).

## Method

We evaluated the benefits of the voice commands for mode and tool change. The implant planning system provided different modes and tools to help the user. There were 15 modes (e.g., switching between gray-scale/colors, use/not use implant handles) and tools (e.g., a selective transparency tool, a tool to align the implant orientations) available in the menu (Fig. 1), with all the labels for reference.

The participants did three implant planning tasks for two implants with two different conditions. In the first condition, *Hand*, mode change and tool selection were made by either pushing a menu button by the controller or pointing it with controller’s ray and pressing a trigger (Fig. 1). In the second condition, *Voice*, voice commands were available and used by saying the name of the mode or tool aloud. That was done by implementing a command-based interface where command utterances, like “xray flashlight” (see Fig. 1), were simply spoken aloud. Having these simple names and a short, known list of possible command expressions ensured a reliable recognition. After recognizing a command, the system provided a generic tone as a confirmation. In case of recognizing a wrong command, the user could simply retry.

**Fig. 2:**
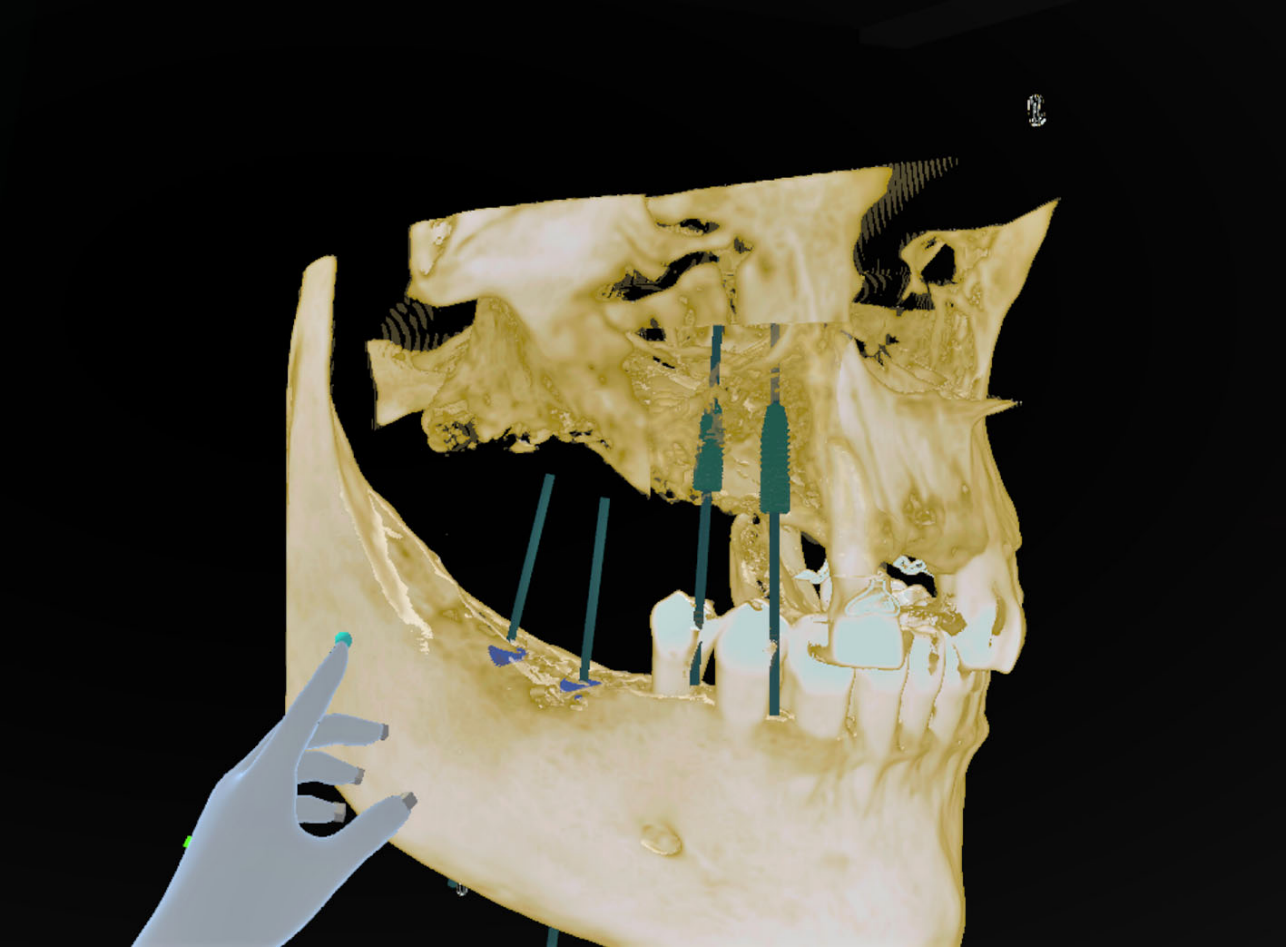
A skull model with four implants planned, one pair on the lower jaw and another pair on the upper jaw. Each pair of implants would support fixed supra-structures

The task was to do the planning of two implants for a dental bridge, see Fig. 2. This was more demanding than finding a location for a single implant as two implants need to be aligned and suitably located. The implants were moved to their planned positions by picking them up using a controller and doing the necessary translations and rotations. Simultaneous coordination of two implants was difficult, they need to be properly positioned in their respective locations individually, but also be in parallel in their final locations.

For the experiment, we prepared a 3D skull model. Skull visualizations were generated in real-time from CBCT DICOM (Digital Imaging and Communications in Medicine standard) volumetric data. Skull model was pseudonymized and may be used in product development with the permission of the individual.

### Measurements

#### Objective measures

We measured the implant placement times from the moment that the planning task was shown to the participant to the moment that the participant released an implant for the last time. We compared the final implant positions between the participants. We also counted the number of implant pickups and releases, and the number of times that the participants were using the voice commands.

#### Subjective measures

We asked the participants to evaluate a number of subjective questions and a statement (see Table 1), using a 7-step Likert scale for the answers from 1 (Not-a-all) to 7 (Very).

**Table 1:** The questions (1 to 5) and a statement (6) that were used to evaluate the subjective impressions of both interaction conditions

1	How successful were you in accomplishing what you were asked to do?
2	How confident you were in your ability to use the interaction method?
3	How efficient was the interaction method to use?
4	How easy was the interaction method to use?
5	Could you imagine using the method for your daily work?
6	I needed to learn a lot of things before I could get going with this system

In the end of the experiment, we asked the participant to select the best condition and give a short reasoning of that specific choice. After each condition and in the end, we offered the participant a chance to give comments and improvement ideas.

## Experiment

**Fig. 3:**
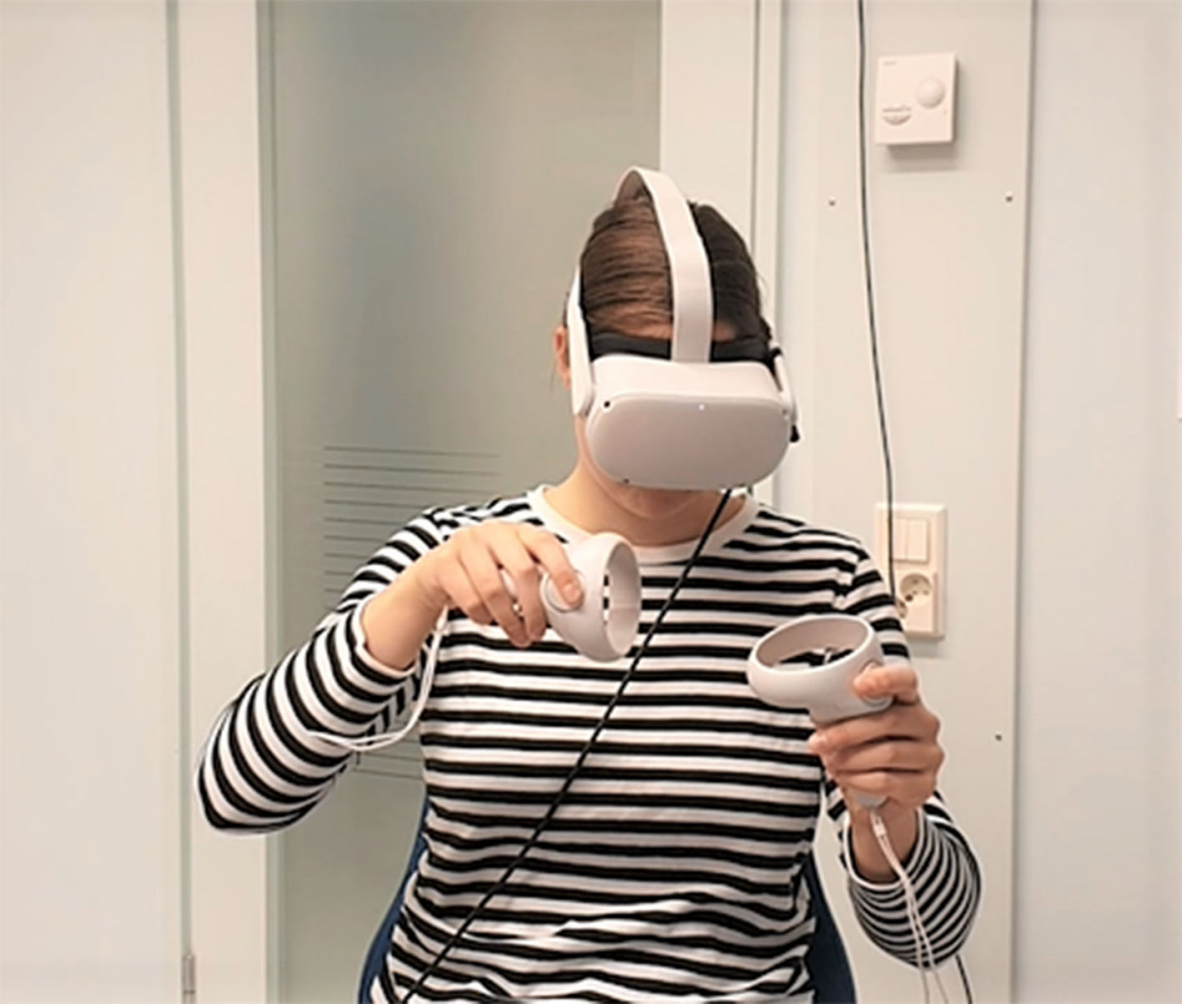
A virtual implant planning tool user with Oculus Quest 2 and controllers

### Apparatus

A VR implant planning implementation by Planmeca company was used as a basis for the experiment software. The experimental system was built using the Oculus Quest 2, Touch controllers (see Fig. 3) and the Unity 3D software development system. For voice recognition, we used the microphone on the Oculus Quest 2 and Microsoft MRTK-Unity (Mixed Reality Toolkit) voice commands. The language of the voice commands was English.

### Participants

We recruited six dentomaxillofacial radiologists who had different amounts of previous experience on the implant planning task using other planning tools. One of the participants did regular implant planning daily, and another did it weekly. The four other participants did implant planning less often.

Previous experience in dentistry varied between 7 years to 36 years. We also asked if and how long experience the participants had of using VR devices and all but one had some experience, but only a few months.

### Procedure

Upon arrival, the participant was introduced to the study and to the equipment. S/he was asked to read and sign a consent form and fill in a background information form.

For both input conditions, the facilitator first demonstrated the system functions and the controls, and then asked the participant to practice the system, at least long enough to be confident to be able to do the tasks. When ready, the participant made three implant plans for the first condition.

The three implant plans were made to the same skull model, to different areas. In Fig. 2, the first and second tasks are done. For all participants, the areas were presented in the same order. The order of conditions was balanced so that three participants did the implant planning first with voice commands. The three others started without voice commands.

After three implant plans had been completed, the participant was asked to fill in a questionnaire to do a subjective evaluation of the condition. After that s/he would repeat the procedure for the second condition. After both conditions had been done, the participant was asked to rank the conditions.

## Results

### Objective measures

The task completion times varied a lot. The completion time minimums were around 45 seconds and the maximums were around 460 seconds, with no systematic trends between the conditions or locations. The median completion time for *Hand* condition was 167.6 seconds with median absolute deviation of 73.2 seconds and for *Voice* condition was 196.0 seconds with median absolute deviation of 67.7 seconds.

**Fig. 4:**
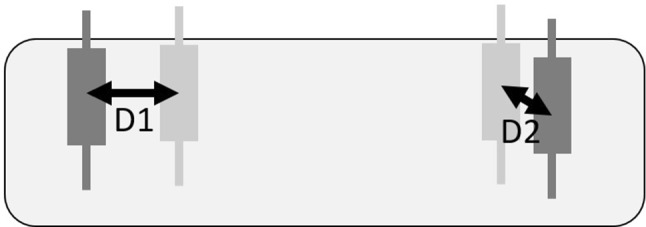
The method of the respective implant distance calculation. For visualization, in one plan the dark gray implants have been planned and in another the light gray implants. The respective distance between the plans was computed by taking the respective implants from each pair, first computing the distances between them (D1, D2), and then a median of these two numbers

**Fig. 5:**
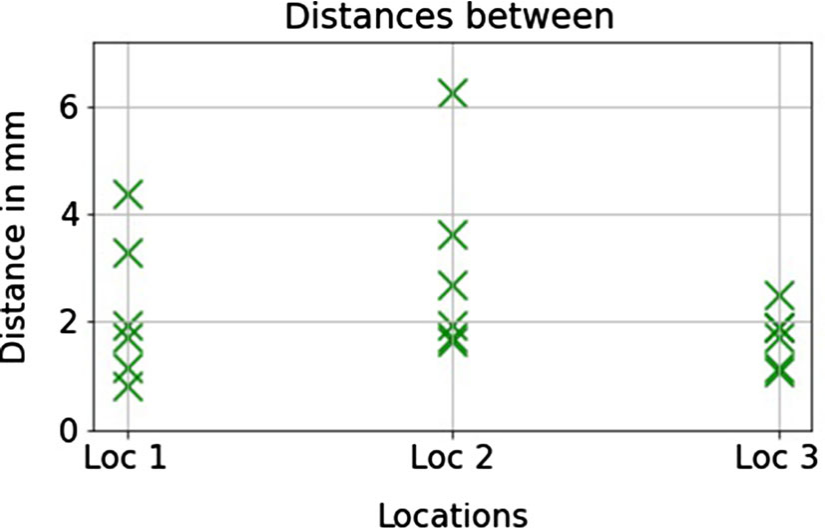
The respective distances between the implants that each participant was setting into the same location (for different conditions). While the maximum distances were slightly over 6 and 4 millimeters, the median distances per location were between 1.8 and 2.3 mm

We recorded all the final positions of all 72 implants that were planned (6 participants times 3 locations times 2 implants per location times 2 conditions). For the analysis of the position consistency, we computed the differences in position when a participant did the same plan twice. As different radiologists have slightly different views of the constraints set by the anatomical features, we often do not have an unambiguous target location for the implant. As two implants were planned in each location we computed the distances between the respective implants and then a median of these (see Fig. 4). The median values of the differences per location (by six participants) were between 1.8 millimeters and 2.3 millimeters, see Fig. 5. As seen in Fig. 2, there was space in the intended area for the implant and variation in locations was expected.

**Fig. 6:**
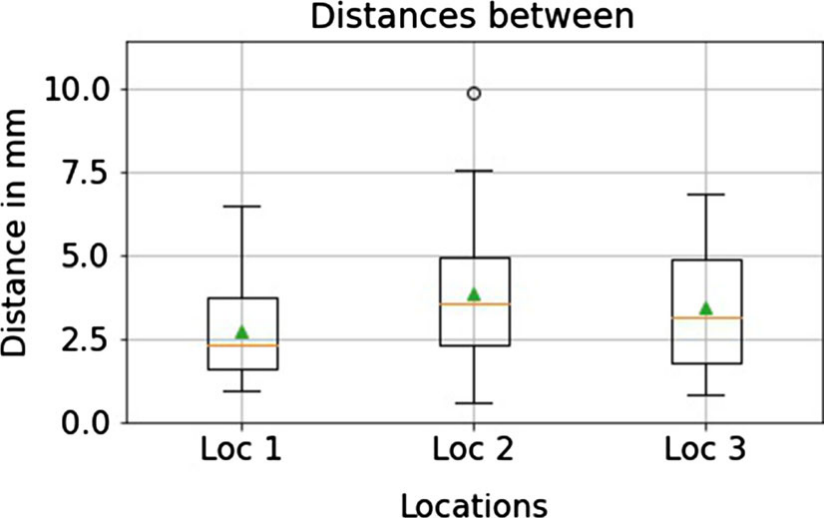
Distances between the respective final implant positions on each location for different participants. The distances are slightly longer than the distances for the same participant (Fig. 5). The median distances per location (the horizontal line) varied between 2.3 millimeters and 3.5 millimeters. The small triangles mark the mean values

For the analysis of the position consistency between participants, we computed the distances between all pairs of respective implants planned in the same location. That meant computing 60 distances (15 participants’ pairwise comparisons times 4 implant comparison pairs) for each location. The median values of the distances per location varied from 2.3 millimeters to 3.5 millimeters; the median absolute deviations varied from 1.0 millimeters to 1.4 millimeters, see Fig. 6.

The numbers of implant pickups varied a lot. The minimum numbers were 4 and 6, while the maximum was around 50. The median pickup numbers for *Hand* condition were 18 times with median absolute deviation of 5.5 times and for *Voice* condition was also 18 times with median absolute deviation of 9 times.

All the participants used the voice commands, while the numbers varied between the participants. The minimum number was 8 times (summed over the three tasks in *Voice* condition) and the maximum number was 45 times; the median value over all participants was 15.5 times. These numbers were quite similar to the use of the button presses for menu selections.

### Subjective measures

**Fig. 7:**
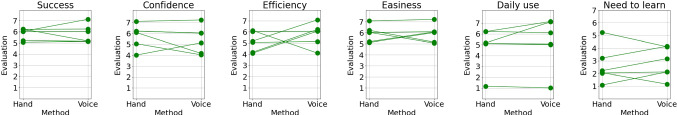
The evaluation results. A line connects the evaluations given by the same participant for both conditions. For most questions, the variation in values is small, but for the last question, there was more variation. For daily use, one participant gave a very different answer than the other participants

In the questionnaires, the participants evaluated the success, confidence, efficiency, and easiness of the condition as well as willingness to use the condition daily and need to learn new things. The results are shown in Fig. 7. Most of the evaluations were positive, and no big differences between the conditions can be seen. One exception is in daily use evaluation, where one participant evaluated the conditions equally weak. Another exception was in the need to learn, where the variation between the evaluations is large for both conditions.

On all measured scales, the conditions got similar, positive evaluations by mean and median values (Table 2). For the last statement (Need to learn), the grade 1 means no need for learning.

In the condition ranking, 3 out of 6 ranked *Voice* more liked and 2 ranked *Hand* more liked. One participant ranked them equally. The ranking results did not correlate with the order of conditions.

## Discussion

One of the recognized challenges in implant planning is the transition between 3D to 2D and back to 3D [3]. The radiologists need to translate the CBCT image on the 2D display to 3D model in their head. This requires spatial understanding and increases the cognitive load [2]. With VR, we can observe the 3D image in 3D environment where the planning can be done. One participant commented that when placing multiple implants, you need to understand the structure of the whole skull and the 3D environment is more understandable than the 2D environment. Due to the differences of 3D and 2D environments, research is needed to understand the requirements of the 3D manipulation tools.

There were no significant differences between the two conditions on completion times. We asked the participants to tell what they are thinking while working with the task to get as much information as possible and some participants spoke more than the others.

There were no significant differences on implant pickup numbers. At least partially that is because of the participants’ way of working in VR affected the number of the pickups.

As there are no unambiguous target locations for the implants, the implant locations slightly varied between the participants and between the two implants that each participant was setting into the same location. The measured variation was expected.

**Table 2:** The median and mean values of the evaluation results for the conditions

		Hand	Voice
Success	Median	6.0	5.5
	Mean	5.7	5.7
Confidence	Median	6.0	5.5
	Mean	5.7	5.3
Efficiency	Median	5.0	6.0
	Mean	5.0	5.7
Easiness	Median	6.0	6.0
	Mean	5.8	5.8
Daily use	Median	5.0	5.5
	Mean	4.7	5.2
Need to learn	Median	2.5	2.5
	Mean	2.8	2.7

There are no clear differences between the conditions for any of the questions

There were no significant differences between the conditions on subjective data from the questionnaire. Both conditions were liked, and the variation was minor. The statement Need of Learn had the most variation. There the variation between the evaluations was wide with both conditions, as some participants found the system to have many issues to learn and to have a big cognitive load, while others found the features familiar.

From the participants’ comments, we can see that the mode change worked well, and it was feasible with both conditions. There were no negative comments and the participants could concentrate on the main task. When using the *Voice* condition, the system recognized the commands on the first or the second attempt.

Half of the participants tried the voice commands accidentally in *Hand* condition as they got used to it. After learning the commands, the use of the system was efficient, reducing the need for reaching for the menu. One participant said that the workflow stayed more intact with voice commands when you did not need to move your hand away from the model. The participants commented that the use of voice commands was natural. However, the hospital might be a noisy environment so that voice commands cannot be used, or it could recognize wrong voices. Privacy requirements or social norms may prevent using voice. The user also needs to memorize the command. It can be concluded that the voice commands need to be short, descriptive, easy to remember, and not too similar. Lupinetti et al. [23] recommended avoiding similar voice commands that can be difficult to remember and easily confused.

In turn, when using hand pointing, the participant was forced to interrupt the main task, look for the menu and the correct button. Then turn back to the implants and continue with the task. We recommend that the future systems would provide both hand pointing and voice commands in parallel so that the participants can choose the interaction modality. While most participants were willing to use the system daily one low evaluation was given by a participant because of VR motion sickness. The motion sickness occurred when turning his/her head from the skull to the implant tray. Motion sickness in VR has been studied to understand the reasons for it [24, 25].

## Conclusions

Six dentomaxillofacial radiologists performed implant planning tasks in virtual reality environment in two conditions, using either only mid-air menu button presses or also voice commands, for tool selection and mode changes. The tool was accepted by the experts and they were able to do multiple-implant planning satisfactorily. While we did not have a formal evaluation of the quality of the planning results, the resulting implant positions were rather similar between the participants, which indicates consistent results. The participants valued the possibility to use the voice commands. Three out of six participants ranked the voice command condition better than the condition without the voice commands, while two ranked the hand pointer better. However, the participants also commented that the use context does not always allow voice use, so other selection options were always necessary. The input methods can be further improved based on the expert comments. Future research for technical and clinical validity of this kind of VR methods is needed.

## Data Availability

The data is available upon request.
